# Retraction Notice to: Long Noncoding RNA UCA1 Regulates PRL-3 Expression by Sponging MicroRNA-495 to Promote the Progression of Gastric Cancer

**DOI:** 10.1016/j.omtn.2022.05.012

**Published:** 2022-05-10

**Authors:** Yi Cao, Jian-Bo Xiong, Guo-Yang Zhang, Yi Liu, Zhi-Gang Jie, Zheng-Rong Li

## Main Text

(Molecular Therapy: Nucleic Acids *19*, 853–864; March 2020)

This article has been retracted at the request of the editors of *Molecular Therapy – Nucleic Acids*.

After concerns were raised by a third party, evidence for image duplication in identical or altered fashion in Figure 6 was identified. This reuse (and in part, misrepresentation) of data without appropriate attribution represents a severe abuse of the scientific publishing system. The corresponding author did not respond when contacted about the retraction.

